# Dynorphin is expressed primarily by GABAergic neurons that contain galanin in the rat dorsal horn

**DOI:** 10.1186/1744-8069-7-76

**Published:** 2011-09-29

**Authors:** Thomas CP Sardella, Erika Polgár, Francesca Garzillo, Takahiro Furuta, Takeshi Kaneko, Masahiko Watanabe, Andrew J Todd

**Affiliations:** 1 Institute of Neuroscience and Psychology, College of Medical, Veterinary and Life Sciences, University of Glasgow, Glasgow G12 8QQ UK; 2 Department of Morphological Brain Science, Graduate School of Medicine, Kyoto University, Kyoto 606-8501, Japan; 3 Department of Anatomy, Hokkaido University School of Medicine, Sapporo 060-8638, Japan

## Abstract

**Background:**

The opioid peptide dynorphin is expressed by certain neurons in the superficial dorsal horn of the spinal cord, but little is known about the types of cell that contain dynorphin. In this study, we have used an antibody against the dynorphin precursor preprodynorphin (PPD), to reveal the cell bodies and axons of dynorphin-expressing neurons in the rat spinal cord. The main aims were to estimate the proportion of neurons in each of laminae I-III that express dynorphin and to determine whether they are excitatory or inhibitory neurons.

**Results:**

PPD-immunoreactive cells were concentrated in lamina I and the outer part of lamina II (IIo), where they constituted 17% and 8%, respectively, of all neurons. Around half of those in lamina I and 80% of those in lamina II were GABA-immunoreactive. We have previously identified four non-overlapping neurochemical populations of inhibitory interneurons in this region, defined by the presence of neuropeptide Y, galanin, parvalbumin and neuronal nitric oxide synthase. PPD co-localised extensively with galanin in both cell bodies and axons, but rarely or not at all with the other three markers. PPD was present in around 4% of GABAergic boutons (identified by the presence of the vesicular GABA transporter) in laminae I-II.

**Conclusions:**

These results show that most dynorphin-expressing cells in the superficial dorsal horn are inhibitory interneurons, and that they largely correspond to the population that is defined by the presence of galanin. We estimate that dynorphin is present in ~32% of inhibitory interneurons in lamina I and 11% of those in lamina II. Since the proportion of GABAergic boutons that contain PPD in these laminae was considerably lower than this, our findings suggest that these neurons may generate relatively small axonal arborisations.

## Background

Laminae I-III of the rat dorsal horn contain a large number of densely packed neurons. Although ~5% of the neurons in lamina I and some of those in lamina III have long ascending axons that project to the brain, the great majority of these cells are interneurons, with axons that arborise locally [1]. The interneurons can be divided into two major classes: excitatory (glutamatergic) cells, and inhibitory cells, which use GABA and/or glycine as their principal fast transmitter [2]. We have reported that GABA-immunoreactivity is present in 25-30% of the neurons in laminae I-II and 40% of those in lamina III, and that a sub-set of these cells show high levels of glycine [3]. Most (if not all) of these inhibitory cells are interneurons. The remaining neurons are glutamatergic, and these include both projection neurons and excitatory interneurons.

Several distinct anti-nociceptive roles have been attributed to inhibitory interneurons in the superficial laminae [4], and in addition, there appears to be a specific population of these cells involved in preventing itch [5]. Less is known about the functions of the excitatory interneurons, although some are thought to transmit information from various types of primary afferent (including nociceptors and low-threshold mechanoreceptors) to projection neurons [6,7].

Numerous studies have attempted to assign the inhibitory and excitatory interneurons in this region to discrete populations based on morphological and/or physiological criteria [1,8-20]. However, although certain characteristic morphological types have been identified [6,10,14-16,21], there is still no generally accepted classification scheme that can account for all of the interneurons in this region [1,9].

An alternative approach to classifying interneurons has been based on their expression of various neurochemical markers, including neuropeptides. Among the many neuropeptides that have been identified in dorsal horn neurons, some (e.g. neurotensin, somatostatin and neurokinin B) are found exclusively in excitatory neurons, some (e.g. galanin and neuropeptide Y; NPY) only in inhibitory neurons, while some (e.g. enkephalin) are expressed by both excitatory and inhibitory cells [22-24].

Studies with immunocytochemistry and *in situ *hybridisation have identified the opioid peptide dynorphin in certain dorsal horn neurons [25-39]. Most of the dynorphin-expressing cells are thought to be interneurons, however, it has been reported that some of those in lamina I are projection cells [30,32,39]. Relatively little is known about the types of neuron that contain dynorphin, although at least some of these are likely to be excitatory interneurons, since some axons containing the peptide were immunoreactive with antibodies against the vesicular glutamate transporter VGLUT2 [35], which is expressed at high levels by excitatory neurons (but not by most primary afferents) in laminae I-III [22,40]. Dynorphin can act at μ, δ and κ opioid receptors [41-43], each of which is expressed in the superficial dorsal horn [44], and also has non-opioid actions that may contribute to neuropathic pain [45].

Dynorphin is normally present at very low levels in the cell bodies of neurons that express the peptide, but these can be revealed with antibody against the precursor protein preprodynorphin (PPD) [39,46], which is also present in dynorphin-containing axon terminals [35]. The initial aims of this study were to determine the proportions of neurons in laminae I-III that contain PPD (and thus express dynorphin) and to test whether any of these cells were GABAergic. Since we found that many of the PPD neurons were GABA-immunoreactive, we also determined the proportion of GABAergic boutons (labelled with antibodies against the vesicular GABA transporter, VGAT) that contain PPD, and looked for coexistence of PPD with other markers of inhibitory interneuron populations [1,47].

## Methods

### Animals

All experiments were approved by the Ethical Review Process Applications Panel of the University of Glasgow, and were performed in accordance with the European Community directive 86/609/EC and the UK Animals (Scientific Procedures) Act 1986.

Spinal cords obtained from 17 adult male Wistar rats (230-330 g; Harlan, Loughborough, UK) were used in this study. The rats were deeply anaesthetised with pentobarbitone (300 mg i.p.) and perfused through the heart with fixative that consisted of either 4% freshly depolymerised formaldehyde (14 rats) or 4% formaldehyde/0.2% glutaraldehyde (3 rats) in phosphate buffer. Tissue fixed with formaldehyde/glutaraldehyde was used to analyse co-localisation of PPD and GABA, since retention of GABA is greatly improved by glutaraldehyde fixation [48,49]. All other parts of the study were carried out on tissue from the rats fixed with formaldehyde.

Mid-lumbar (L3-L5) spinal segments were dissected out and stored in the corresponding fixative for between 5 and 24 hours. These were then rinsed and cut into 60 μm thick transverse sections with a Vibratome. The sections were treated with 50% ethanol for 30 mins to enhance antibody penetration, and those from animals that had been fixed with formaldehyde/glutaraldehyde were incubated with 1% sodium borohydride for 30 mins.

In several parts of the study, a tyramide signal amplification (TSA) method was used, in order to minimise the amount of primary antibody required. All immunocytochemical reactions were carried out at 4°C. For those involving TSA, antibodies were diluted in PBS that contained 0.15 M NaCl, together with the blocking serum supplied by the manufacturer. For reactions that did not involve TSA, antibodies were diluted in PBS that contained 0.3 M NaCl, and blocking serum was not used. Incubations in primary and secondary antibodies were for 3 days and overnight, respectively. Species-specific secondary antibodies (anti-IgG) were raised in donkey and conjugated to Alexa 488 (Invitrogen, Paisley, UK) or to Rhodamine Red, Cy5 or DyLight 649 (Jackson Immunoresearch, West Grove, PA, USA). These were used at 1:500 (Alexa 488 and DyLight 649 conjugates) or 1:100 (Rhodamine Red and Cy5 conjugates). Control experiments were carried out for the TSA reaction by omitting primary antibody, and these showed no immunostaining.

### Proportion of neurons in laminae I-III that were PPD-immunoreactive

Sections from the L4 segments of 3 rats fixed with formaldehyde were incubated in guinea pig antibody against PPD [46] (1:5,000) and mouse monoclonal antibody NeuN (Millipore, Watford, UK, catalogue number MAB377; 1:500). The PPD was revealed with a TSA reaction (tetramethylrhodamine kit; PerkinElmer Life Sciences, Boston, MA, USA) and NeuN with secondary antibody conjugated to Cy5. The sections were then incubated in Sytox Green (Invitrogen; 1:50,000) for 30 mins at 20°C to reveal cell nuclei [24].

Two sections from each of the 3 animals were selected (before PPD immunostaining was viewed) and scanned with a Zeiss LSM710 confocal microscope with Argon multi-line, 405 nm diode, 561 nm solid-state and 633 nm HeNe lasers. The sections were scanned through a 40× oil-immersion objective lens (numerical aperture [NA] 1.3) to produce z-series consisting of 24 optical sections at 1 μm z-separation. Between 6 and 8 contiguous fields were scanned in order to cover the entire cross-sectional area of laminae I-III. The confocal scans were analysed with a modification of the disector method [50-52], as described previously [53,54]. The channels representing NeuN and Sytox were initially opened in Neurolucida for Confocal software (MicroBrightField, Colchester, VT, USA), and an outline of the grey matter was drawn from maximum intensity projections. The 14th and 22nd optical sections in each z-series were designated as the reference and look-up sections, respectively. Every section in each series was then viewed, and neuronal nuclei (identified by the presence of both NeuN and Sytox staining) that were present in the reference section, or appeared in subsequent sections, were marked on the drawing. All of those with nuclei that were still present in the look-up section were then excluded, leaving only neurons for which the lower surface of the nucleus lay between reference and look-up sections. The depth within the z-series at which the nucleus of each of these selected cells was maximal was recorded, to allow subsequent comparison of the depths of PPD^+ ^and PPD^- ^neurons [54]. The channel corresponding to PPD-immunostaining was then viewed, and the presence or absence of PPD immunoreactivity in each of the selected cells was noted. The borders between laminae I, II and III were identified by overlaying images obtained with a dark-field condensor, in which lamina II can be recognised as a dark band due to its lack of myelin [54,55]. The ventral border of lamina III was drawn from a rat spinal cord atlas [56]. Since the frequency of PPD-immunoreactive neurons appeared to differ considerably between the inner and outer parts of lamina II, we divided lamina II by drawing a line midway between its dorsal and ventral borders. In this way, we were able to determine the proportions of neurons in each lamina (and in the dorsal and ventral halves of lamina II) that were PPD-immunoreactive. Since the number of lamina I neurons included in these sections was relatively low (15-30 per section), we also scanned lamina I in an additional section from each of the 3 animals. These were analysed in the same way, except that only neurons in lamina I were included. Although the reference and look-up sections are set relatively far apart with this method, we were able to ensure that all neurons with nuclei that lay between these two planes were included in the sample, by examining every optical section between them. The positions of reference and look-up sections within the z-series were chosen to ensure that perikaryal cytoplasm at both poles of the selected cells was visible in all cases. This was done so that even cells with weak PPD staining would be recognised.

### PPD expression by GABA-immunoreactive neurons

Sections from the L4 segments of the 3 rats that were fixed with formaldehyde/glutaraldehyde were incubated in anti-PPD (1:100) and rabbit antibody against GABA [57] (1:5,000), and these were revealed with secondary antibodies conjugated to Rhodamine Red and Alexa 488, respectively. Six sections from each animal were selected, before immunostaining was viewed, and these were scanned with the confocal microscope through a 40× oil-immersion lens. For each section 6-8 adjacent z-series, each with 1 μm z-separation, were scanned in such a way as to cover the entire area of laminae I-IV on one side of the spinal cord. Since penetration of the GABA-immunostaining was extremely limited, only the superficial parts of each section were scanned.

The confocal images were analysed with Neurolucida for Confocal. The channel corresponding to PPD was initially viewed, and all PPD-immunoreactive neurons for which part of the nucleus appeared at the top surface of the Vibratome section were plotted onto an outline of the dorsal horn. The GABA channel was then revealed, and the presence or absence of GABA-immunoreactivity in each of the selected neurons was recorded. In order to determine whether differences in the sizes of GABA^+ ^and GABA^- ^neurons could have led to a bias in selection, we estimated the distance in the z-axis between the upper surface of the Vibratome section and the bottom of the nucleus for each cell, by counting the number of optical sections between these levels.

### Proportion of GABAergic boutons that were PPD-immunoreactive

Antibody against VGAT was used to reveal GABAergic axonal boutons [58-60]. VGAT (which is sometimes referred to as the vesicular inhibitory amino acid transporter) is also expressed by glycinergic axon terminals [58]. However, most glycinergic axons in laminae I-III are likely to use GABA as a co-transmitter, since virtually all of the glycine-enriched neurons in this region are GABA-immunoreactive [2]. For simplicity, we refer to VGAT-positive structures as GABAergic. It is unlikely that a significant number of GABAergic/glycinergic boutons in this region lack detectable VGAT, since we have shown that 97% of gephyrin-immunoreactive profiles in laminae I-III of the rat dorsal horn are associated with a VGAT-positive bouton [61], and gephyrin is thought to be present at the post-synaptic aspect of most (if not all) GABAergic and glycinergic synapses [62].

Sections from 3 rats that had been fixed with formaldehyde were incubated in guinea pig anti-PPD (1:100), mouse monoclonal anti-VGAT (Synaptic Systems, Göttingen, Germany; catalogue number 131 011; 1:1,000) and rabbit antibody against protein kinase Cγ (PKCγ; Santa Cruz Biotechnology, Santa Cruz, CA, USA; catalogue number sc-211; 1:1,000). These were revealed with secondary antibodies conjugated to Alexa 488, Cy5 and Rhodamine Red, respectively. Two sections from each rat were selected (before PPD immunostaining was viewed) and scanned with the confocal microscope through a 63× oil-immersion lens (NA 1.4) to produce a set of 4 contiguous z-stacks (each consisting of 25 optical sections at 0.3 μm z-separation) that formed a 100 μm wide vertical strip through laminae I-III in the central part of the dorsal horn on one side. The confocal images were analysed with Neurolucida for Confocal software, as described previously [47,54,61]. Briefly, the image stacks were aligned and the outline of the dorsal horn, together with laminar boundaries, was drawn from scans obtained with a dark-field condensor [55] and from the location of the dense plexus of PKCγ-immunoreactivity, which occupies the inner half of lamina II [63]. The VGAT channel was initially viewed, and 300 immunoreactive boutons (100 from each of laminae I, II and III) were selected from a single optical section near the middle of the z-series. The selection was performed by applying a 5 μm × 5 μm grid and choosing the bouton nearest the lower right hand corner of each grid square, starting at the most dorsal part of the lamina and working from dorsal to ventral, and then from left to right. Once the boutons had been selected, the PPD channel was viewed, and the presence or absence of PPD-immunostaining in each VGAT bouton was recorded. Since this sampling method will be biased towards boutons that are more extensive in the z-axis, we measured the z-axis length of all selected boutons by multiplying the number of z-sections on which they appeared by the z-separation between optical sections (0.3 μm).

### Relationship between PPD and markers of inhibitory interneurons

We have previously shown that NPY, galanin, neuronal nitric oxide synthase (nNOS) and parvalbumin are present in 4 distinct, non-overlapping populations of inhibitory interneurons in the superficial dorsal horn of the rat spinal cord [47,64]. In order to determine whether dynorphin was expressed by any of these populations, we reacted sections from the L3-L5 segments with guinea pig anti-PPD (1:5,000) and either rabbit anti-NPY (Bachem, St Helens, UK; catalogue number T-4070; 1:1,000), rabbit anti-galanin (Bachem, St Helens, UK; catalogue number T-4334; 1:1,000); rabbit anti-parvalbumin [65] (1.24 μg/ml) or sheep anti-nNOS [66] (1:2,000). In each case PPD was revealed with a TSA method (as described above) and the other antigen with a secondary antibody conjugated to Alexa 488.

Sections from 3 rats that had been reacted with each of these antibody combinations were scanned with the confocal microscope through a 40× oil-immersion objective to produce a set of z-series (2 μm z separation) that covered the entire area of lamina I and II on one side. Two sections were scanned from each animal for each combination, and these were analysed with Neurolucida. In each case, the channel corresponding to PPD was viewed first, and the positions of all immunoreactive neurons in laminae I and II were recorded. The other channel was then viewed, and the presence or absence of the other marker (NPY, galanin, parvalbumin or nNOS) was recorded. Since we found a high degree of co-localisation of PPD with galanin (see below), we also identified all of the neurons that were galanin-immunoreactive but lacked PPD, and in this case, we analysed cells in laminae I and II separately.

To test for co-localisation of PPD and galanin in GABAergic axonal boutons, we reacted sections from the L4 or L5 segments of 3 rats with guinea pig anti-PPD (1:100), rabbit anti-galanin (1:1,000) and goat anti-VGAT [67] (1:5,000), and these were revealed with secondary antibodies conjugated to Rhodamine Red, Alexa 488 and DyLight 649, respectively. A single section from each animal was scanned with the confocal microscope through the 63× oil-immersion lens, exactly as described above (for PPD, VGAT and PKCγ), except that only laminae I and II were included in the scans. The resulting z-series were analysed with Neurolucida. In each case, the PPD and VGAT staining were initially viewed, and 100 PPD^+^/VGAT^+ ^boutons were selected from the superficial dorsal horn in each section. The galanin staining was then viewed, and the presence or absence of galanin-immunoreactivity in each of the selected boutons was recorded. The analysis was then repeated in the same way, except that 100 galanin^+^/VGAT^+ ^boutons were selected from each section and then examined for the presence of PPD.

### Characterisation of antibodies

The PPD antibody was raised against a peptide corresponding to amino acids 229-248 at the C terminus of rat preprodynorphin, which was conjugated to keyhole limpet haemocyanin. It has been shown to label PPD, but not dynorphin or enkephalin, and tissue staining is blocked by pre-absorption with the immunising peptide [46].

The monoclonal antibody NeuN, raised against purified cell nuclei from mouse brain, detects a protein specific to neurons [68], and we have reported that it appears to label all neurons (and no glial cells) in the rat spinal cord [55]. The GABA antibody was raised against GABA conjugated with glutaraldehyde to porcine thyroglobulin, and shows negligible cross-reactivity against other amino acids, including glutamate, aspartate, glycine or taurine [57]. We have shown that immunostaining with the NPY antibody in the dorsal horn can be abolished by pre-incubation with synthetic NPY [69]. The nNOS antibody labels a band of 155 kDa in Western blots of rat hypothalamus, and immunostaining is abolished by pre-incubation in nNOS [66]. The parvalbumin antibody was raised against mouse parvalbumin and recognises a single band of 13 kDa in blots of mouse brain homogenates [65]. The galanin antibody detects rat galanin, but not substance P, vasoactive intestinal polypeptide or NPY (manufacturer's specification), and it has been reported that staining with this antibody is absent from the brains of galanin knock-out mice [70]. The mouse monoclonal VGAT antibody (raised against amino acids 75-87 of mouse VGAT) labels a single band of 57 kDa in blots of mouse brain and retina, and immunostaining is blocked by pre-absorption with the immunising peptide [71]. No staining with this antibody is seen in Western blots from cultured neurons obtained from VGAT^-/- ^mice [72]. The goat VGAT antibody (raised against amino acids 21-112 of mouse VGAT) also stains a single band of 57 kDa in blots of brain extracts, and we have found that this stains identical structures in rat dorsal horn to the mouse VGAT antibody (E. Polgár and A.J. Todd, unpublished observations). We have reported that the rabbit PKCγ antibody stains identical structures to a well-characterised guinea-pig antibody [61], and staining with the latter antibody is absent from the brains of PKCγ knock-out mice [73].

### Statistical tests

The Mann-Whitney U-test was used for statistical analysis, and p < 0.05 was taken as significant.

## Results

### PPD immunoreactivity in laminae I-III

**Figure 1:**
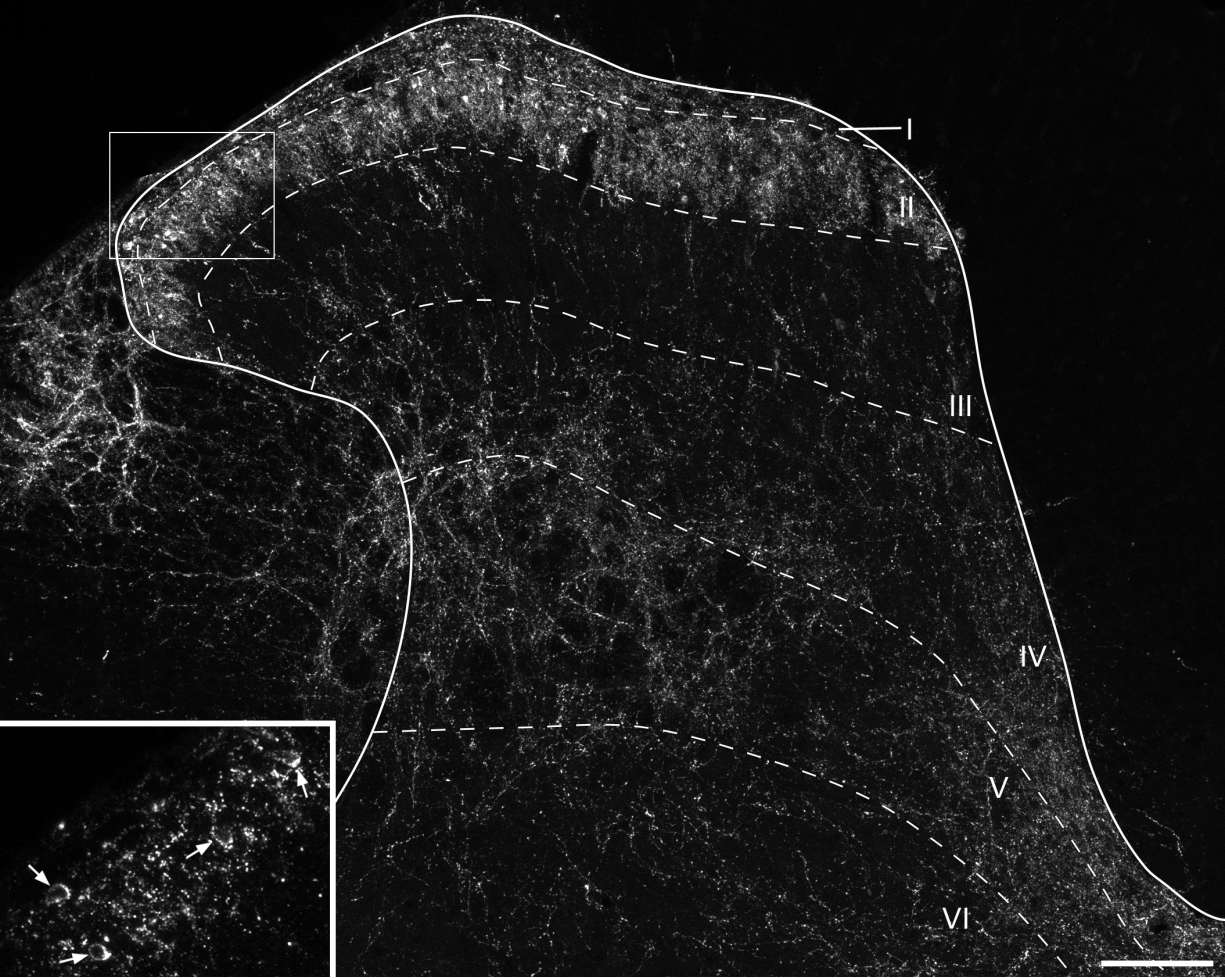
**PPD immunoreactivity in the rat dorsal horn**. A confocal image from a transverse section of the L4 segment showing the laminar distribution of PPD. There is a dense band of immunostaining that occupies lamina I and lamina IIo, with variable extension into lamina IIi. This region contains several immunoreactive cell bodies, which are seen more clearly in the inset (corresponding to the box in the main image). Four immunoreactive cells are indicated with arrows. Lamina III contains a few scattered immunoreactive axons, while these are somewhat more numerous in laminae IV-V. The main image is a projection of 27 optical sections, while the inset is a projection of 2 sections. In each case the z-spacing is 2 μm. Scale bar = 100 μm

**Figure 2:**
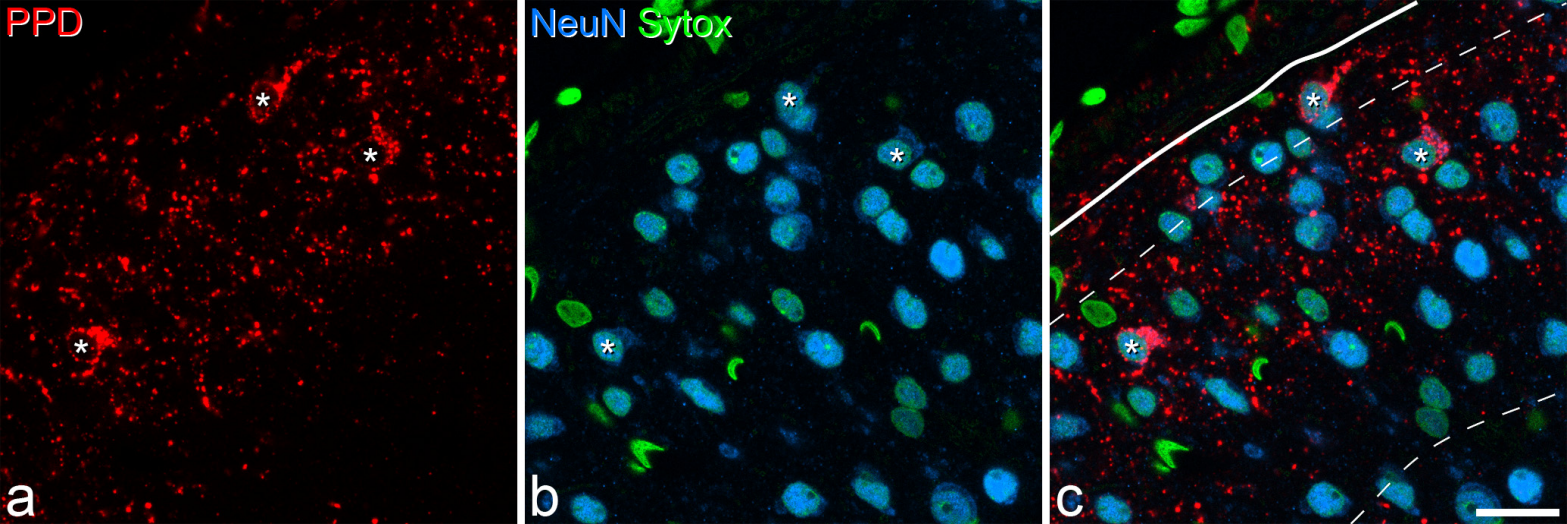
**Confocal images showing PPD-immunoreactive cells in lamina I and IIo**. **a: **Part of the L4 segment scanned to reveal PPD (red). **b**: The same field scanned for NeuN (blue) and Sytox (green). **c**: Merged image, with the solid line representing the dorsal border of the grey matter, and the two dashed lines the dorsal and ventral borders of lamina II. Three PPD-immunoreactive cells (asterisks) are present, one in lamina I and 2 in lamina IIo. Note that these are stained with NeuN, indicating that they are neurons. Many other neurons that are not PPD-immunoreactive are also visible. The nuclei of non-neuronal cells lack NeuN, and therefore appear green. The small red profiles include PPD-immunoreactive axons and dendrites. The images were obtained from a single optical section. Note that some neuronal nuclei appear very small, because the optical section does not go through their maximum diameter. Scale bar = 20 μm.

The laminar distribution of PPD immunoreactivity was consistent with that which has been reported previously in the rat spinal cord with antibodies against dynorphin [28,33,35] or PPD [35,37]. Axonal immunostaining, which consisted of fibres and associated varicosities, was present throughout the dorsal horn (Figure 1). These axons formed a dense plexus in lamina I and the outer part of lamina II (lamina IIo), and were also fairly numerous throughout the deepest parts of the dorsal horn (laminae IV-VI). However, there were relatively few PPD-immunoreactive axons in the inner part of lamina II (lamina IIi) or in lamina III. PPD-immunoreactive cell bodies were frequently seen in the superficial region (laminae I-IIo), but only occasionally in deeper laminae. Within these cells, the immunoreactivity was present in the form of numerous fluorescent granules that occupied the perikaryal cytoplasm, with limited extension into proximal dendrites (Figure 2a). PPD-immunoreactive profiles (both axons and cell bodies) were seen throughout the depth of the Vibratome sections, indicating that there was good penetration of antibodies into the tissue.

The results of the quantitative analysis of PPD expression in neurons in laminae I-III are shown in Table 1, and an example of PPD and NeuN immunostaining in Figure 2. PPD was present in 17.1% of lamina I neurons, 4.3% of those in lamina II and 0.7% of those in lamina III. When lamina II was divided into outer and inner halves, the proportions of neurons in these that were PPD-immunoreactive were 7.6% and 0.4%, respectively. The depths at which the nuclei of the PPD^+ ^cells in the sample were maximal ranged from 5 to 14 μm below the surface (median 10 μm, n = 65), while the corresponding values for PPD^- ^cells were 2 to 21 μm (median 11 μm, n = 1196), and these values did not differ significantly (p = 0.12; Mann-Whitney U-test). This suggests that there was no reduction in the proportion of neurons that were PPD-immunoreactive at deeper levels within the z series, which would have occurred if there had been incomplete penetration of immunostaining.

**Table 1:** Percentages of neurons in laminae I-III that were PPD-immunoreactive

Lamina	Number of neurons counted	Number of PPD^+ ^cells	% of neurons that were PPD^+^
I	68.7 (60-76)	11.7 (11-13)	17.1 (15.7-18.3)

II	205.7 (183-221)	9 (7-10)	4.3 (3.8-4.7)

IIo	113.3 (105-129)	8.7 (6-10)	7.6 (5.7-9.5)

IIi	92.3 (77-108)	0.3 (0-1)	0.4 (0-1.3)

III	146 (127-170)	1 (0-2)	0.7 (0-1.4)

In each case the mean values for the 3 animals are shown, with the range in brackets.

### PPD and GABA

**Figure 3:**
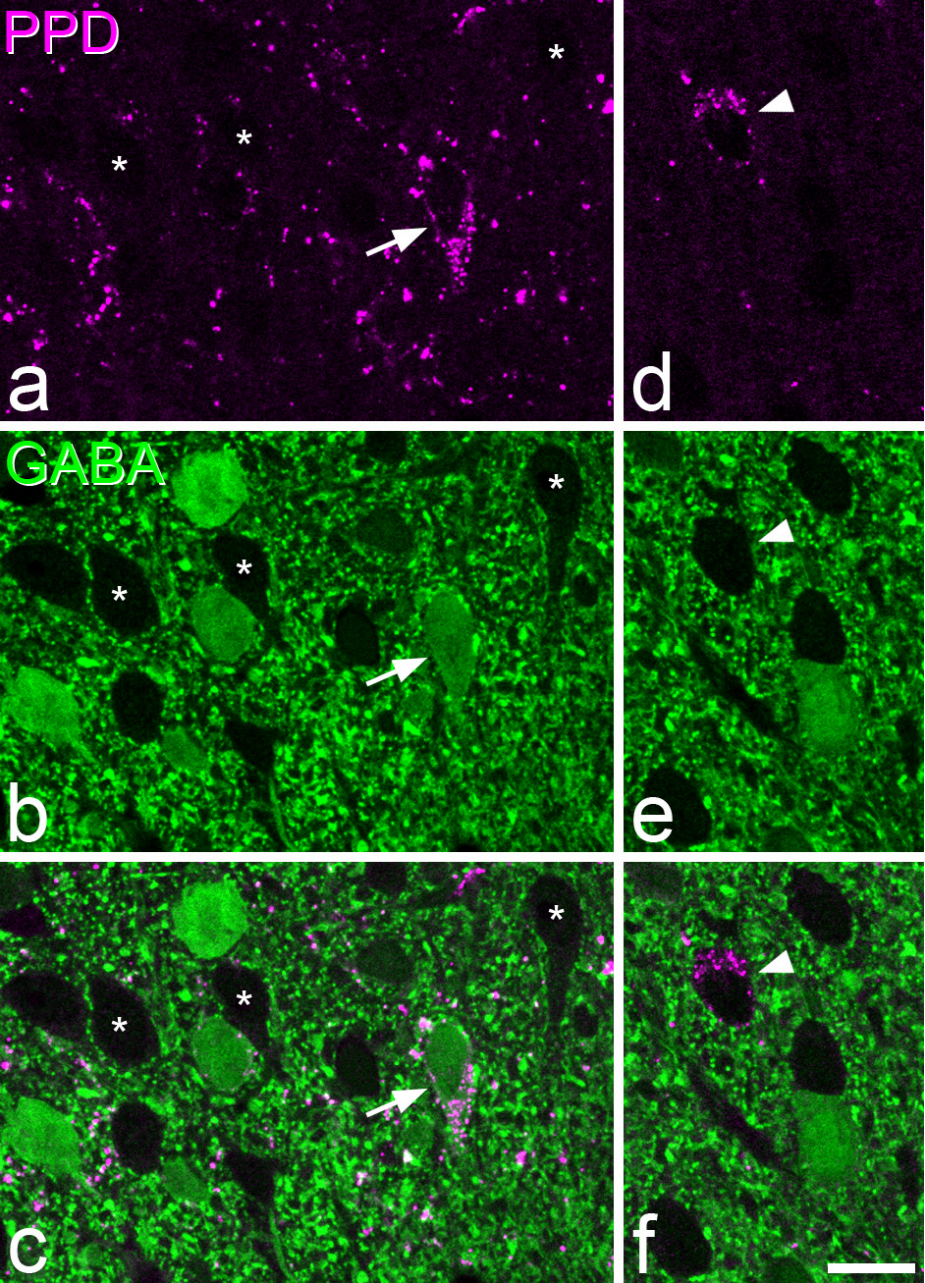
**Expression of PPD by GABA-immunoreactive and non-immunoreactive neurons**. **a**-**c**: Confocal images of a transverse section of the L4 segment from a rat that had been fixed with glutaraldehyde/formaldehyde and reacted with antibodies against PPD (magenta) and GABA (green). This field shows a single PPD-immunoreactive neuron in lamina IIo (arrow), which is GABA-immunoreactive and is surrounded by other GABA-immunoreactive neurons, as well as by neurons that lack GABA, three of which are indicated with asterisks. Note that the PPD occupies the perikaryal cytoplasm but is excluded from the nucleus, whereas GABA staining is seen throughout the nucleus and cytoplasm. Also, note that there is a high density of punctate GABA immunoreactivity throughout the neuropil, which consists of the dendrites and axons of GABAergic neurons. The GABA-negative neuronal cell bodies stand out as dark "holes" within this neuropil staining. **d**-**f**: a PPD-immunoreactive neuron (arrowhead) near the lamina II/III border that is not GABA-immunoreactive. Both sets of images are from a single optical section. Scale bar = 10 μm.

**Figure 4:**
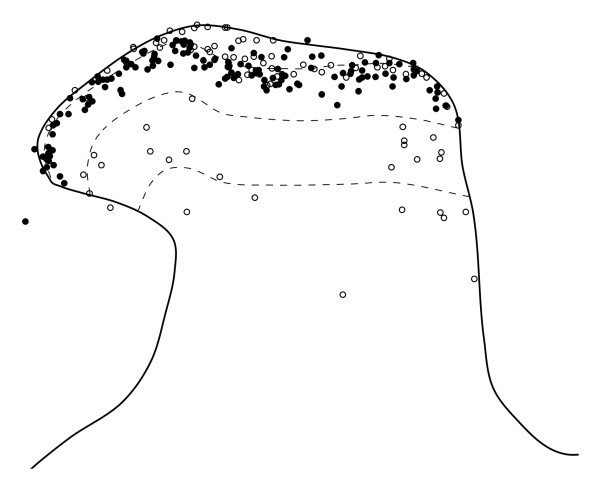
**The laminar location of GABA-immunoreactive and non-immunoreactive PPD neurons**. A plot showing the distribution of all of the PPD cells that were analysed for GABA-immunoreactivity in the three rats that were fixed with glutaraldehyde/formaldehyde. Each symbol represents a single PPD neuron. The GABA-immunoreactive cells are shown as filled circles and the GABA-negative cells as open circles. Note that while the majority of cells (143/208) in the superficial dorsal horn (laminae I-II) are GABA-immunoreactive, all of the PPD cells ventral to this region are GABA-negative.

The distribution of GABA immunostaining was very similar to that reported previously in the rat dorsal horn [2,3]. Staining was particularly dense in laminae I-III, and this region contained numerous immunoreactive cell bodies (Figure 3). Non-immunoreactive cells stood out as dark areas, surrounded by strongly immunoreactive structures in the neuropil, which presumably corresponded to dendrites and axonal boutons of GABAergic neurons. However, as reported previously for Vibratome sections [61,74], the penetration of GABA immunostaining was extremely limited (less than 5 μm), and we therefore only examined cells for which the nucleus appeared on the top surface of the section. As in the formaldehyde-fixed tissue, numerous PPD-immunoreactive cells were present in laminae I-II, with scattered cells in deeper laminae. Examples of PPD cells that were GABA-immunoreactive, or non-immunoreactive are illustrated in Figure 3, while the locations of all the PPD cells tested for GABA-immunoreactivity are shown in Figure 4, and quantitative data for those in laminae I-III are provided in Table 2. The majority of the PPD cells that were selected in laminae I-II (143/208, 69%, data pooled from 3 rats) were GABA-immunoreactive, while all of the cells in lamina III and below were negative for GABA. Within the superficial dorsal horn, GABA-immunoreactivity was seen in 47% of PPD cells in lamina I and 79% of those in lamina II.

**Table 2:** Percentages of PPD neurons sampled in laminae I-III that were GABA-immunoreactive

Lamina	Number of PPD cells counted	% that were GABA^+^
I	22.7 (17-28)	46.6 (39.1-53.6)

II	46.7 (42-51)	79.1 (73.8-85.1)

III	5.7 (5-7)	0

In each case the mean values for the 3 animals are shown, with the range in brackets.

Since both GABA^+ ^and GABA^- ^PPD cells were found in laminae I-II, we compared the distances between the top of the Vibratome section and the bottom of the nucleus for all of the cells in both populations within this region. Because each nucleus can be cut through any part of its z-axis length, the mean of this distance should correspond to half of the mean z-axis length of the nucleus in a large population of cells. The mean distance was 7.26 μm (range 1-15 μm, median 7 μm, n = 143) for the GABA^+ ^neurons, and 8.17 μm (range 1-14 μm, median 9 μm, n = 65) for the GABA^- ^cells. These values did not differ significantly (p = 0.074, Mann-Whitney U-test), which suggests that bias towards selecting larger cells was unlikely to have influenced our estimate of the proportion of PPD neurons that were GABA-immunoreactive.

### PPD and VGAT

**Figure 5:**
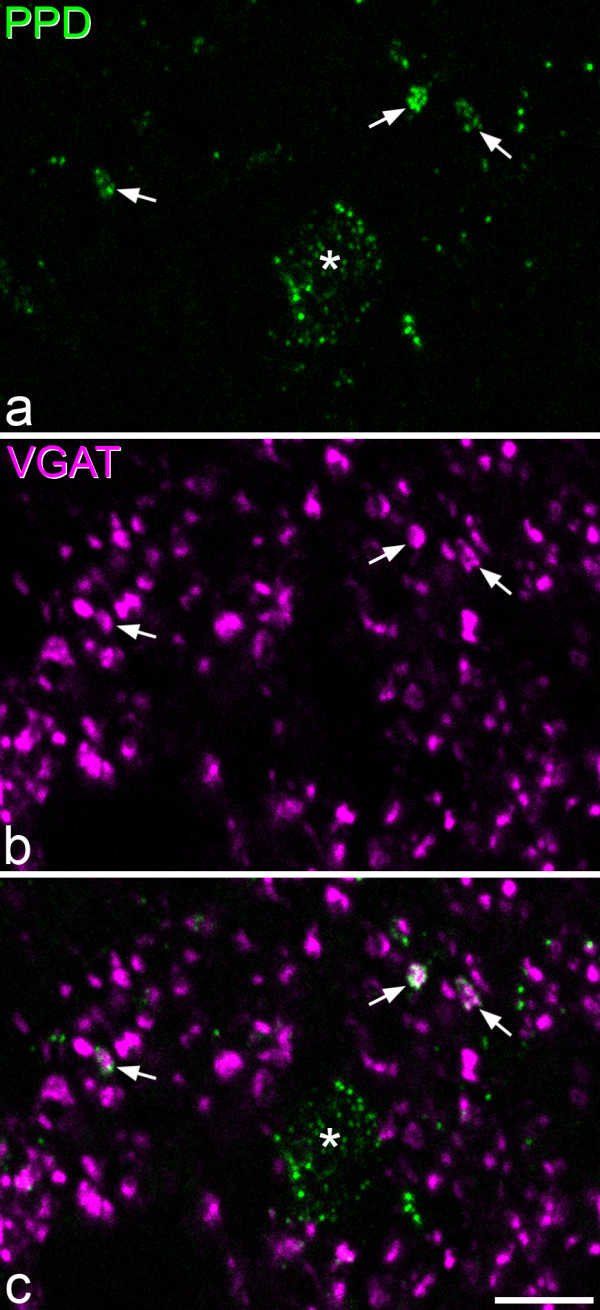
**PPD and VGAT immunoreactivity in lamina I**. **a**, **b**: Confocal images scanned to reveal PPD (green) and VGAT (magenta). **c**: a merged image. Many VGAT boutons are visible throughout the field, and 3 of these (arrows) are also PPD-immunoreactive. The asterisk in **a **and **c **indicates part of a PPD^+ ^cell body. The images are projections from 3 optical sections at 0.3 μm z-separation. Scale bar = 5 μm.

In the sections reacted for VGAT and PPD, the distribution of VGAT-immunostaining was the same as that reported previously [54], with numerous VGAT-immunoreactive boutons being present throughout the entire dorsal horn. PPD-immunoreactivity was found in some of the VGAT boutons (Figure 5), particularly in laminae I and IIo. Quantitative analysis revealed that 4.2% of the VGAT boutons in both lamina I and lamina II were positive for PPD, but very few (0.2%) of those in lamina III were PPD-immunoreactive (Table 3). When lamina II was subdivided into outer and inner halves, the proportions of VGAT boutons that were PPD^+ ^were 6.8% in IIo and 2.1% in IIi.

**Table 3:** Percentages of VGAT boutons in laminae I-III that were PPD-immunoreactive

Lamina	VGAT boutons analysed	% VGAT boutons PPD^+^
**I**	600 (200)	**4.2 **(3 - 5.5)

**II**	600 (200)	**4.2 **(3 - 5)

**IIo**	267 (87-90)	**6.8 **(4.4 - 9.2)

**IIi**	333 (110-113)	**2.1 **(1.8 - 2.7)

**III**	600 (200)	**0.2 **(0 - 0.5)

The second column shows the total number of VGAT boutons from each lamina (or sublamina) that were analysed, with the range per animal (n = 3 rats) shown in brackets. The mean percentages of the VGAT-positive boutons that were PPD-immunoreactive are shown in the third column, with ranges per animal in brackets.

The mean of the z-axis lengths for the PPD^+ ^boutons in laminae I, IIo and IIi were 1.67 μm (range 0.6-2.4 μm, n = 25), 1.68 μm (range 1.2-2.7 μm, n = 18) and 1.63 μm (range 1.5-2.1 μm, n = 7), respectively, while those for the PPD^- ^boutons in these laminae were 1.59 μm (range 0.3-3.6 μm, n = 575), 1.48 μm (range 0.3-3.6 μm, n = 249) and 1.48 μm (range 0.3-3.3 μm, n = 326). Mann-Whitney U-tests showed that there was no significant difference between the sizes of the PPD^+ ^and PPD^- ^VGAT boutons in any of these regions (p = 0.28 for lamina I, 0.09 for lamina IIo, 0.26 for lamina IIi). It is therefore unlikely that our sample was biased towards either PPD^+ ^or PPD^- ^boutons among the VGAT population.

### Colocalisation of PPD with nNOS, parvalbumin, NPY and galanin

**Figure 6:**
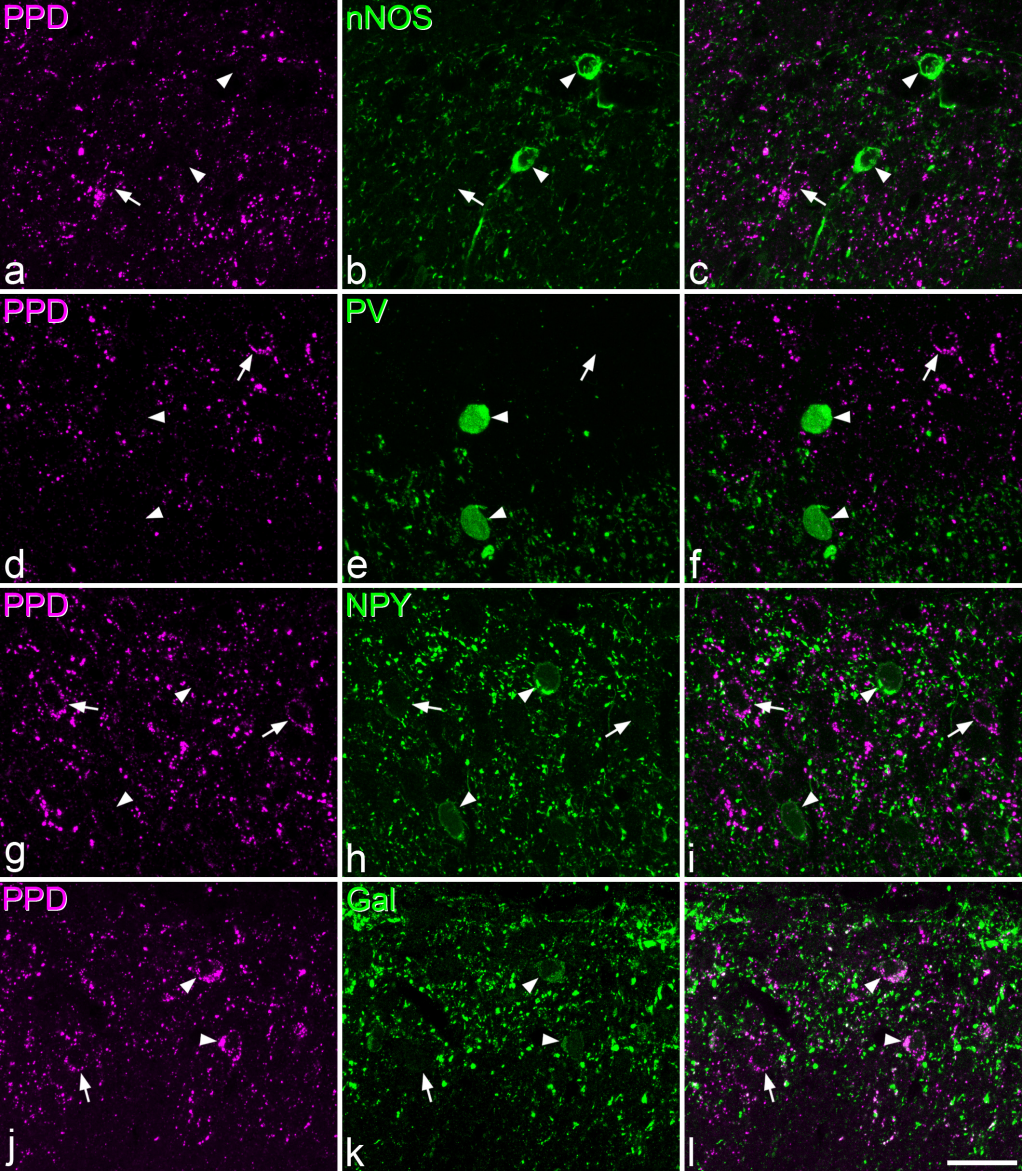
**The relationship between PPD, nNOS, parvalbumin, NPY and galanin**. Confocal scans from the superficial dorsal horn to show staining for PPD and four other compounds that have been detected in subsets of inhibitory interneurons: nNOS, parvalbumin (PV), NPY and galanin (Gal). In each case the image on the left shows PPD (magenta), the one in the middle shows the other compound (green), while the one on the right is a merged image. **a**-**c**: a scan through lamina I and the outer part of lamina II shows a PPD-immunoreactive cell (arrow) close to 2 nNOS^+ ^cells (arrowheads). **d**-**f**: a scan through the middle part of lamina II shows a PPD^+ ^cell (arrow) and two parvalbumin cells (arrowheads). Note that the upper part of this field (lamina IIo) contains numerous PPD-immunoreactive profiles and little parvalbumin staining, while the lower part (lamina IIi) has many parvalbumin-containing structures, and relatively few that are PPD^+^. **g**-**i**: a field from lamina II that contains two PPD cells (arrows) and two NPY cells (arrowheads). Note the lack of co-localisation of PPD with nNOS, parvalbumin or NPY in these images. **j**-**l**: a scan through laminae I and IIo shows 3 PPD cells. Two of these (arrowheads) are also galanin-immunoreactive, while the other one (arrow) is not. Images were obtained from 3 (a-f) or 4 (g-l) confocal images at 0.5 μm z-spacing. Scale bar = 20 μm.

The distribution of nNOS, parvalbumin, galanin and NPY was identical to that reported previously in the rat dorsal horn with these antibodies [47]. Results of the quantitative analysis are provided in Tables 4 and 5, and confocal images showing the relationship between PPD and each of these compounds are illustrated in Figure 6.

**Table 4:** Lack of co-localisation of PPD with nNOS, parvalbumin and NPY in laminae I-II

	Number of PPD cells examined	Number positive for other marker	% positive for other marker
nNOS	79 (68-92)	0	0

Parvalbumin	108 (87-129)	0	0

NPY	102.7 (92-113)	7 (4-10)	6.8 (3.9-8.8)

In each case the mean values for the 3 animals are shown, with the range in brackets.

**Table 5:** Co-localisation of PPD with galanin

Lamina	Number of PPD cells	Number of galanin cells	Number of double-labelled cells	% of PPD cells with galanin	% of galanin cells with PPD
I	43 (38-46)	20.7 (19-24)	20 (18-24)	46.6 (39.1-53.3)	96.5 (94.7-100)

II	89.3 (70-108)	67.3 (55-74)	61.7 (54-68)	70 (63-77.1)	92.1 (86.3-98.2)

III-IV	12 (8-17)	6 (4-9)	0	0	0

In each case the mean values for the 3 animals are shown, with the range in brackets.

In sections reacted with nNOS and PPD antibodies there was some overlap in the laminar distribution of cells with the two types of immunoreactivity. However, none of the 237 PPD^+ ^cells selected was nNOS-immunoreactive (Figure 6a-c). Parvalbumin-immunoreactive cells were largely restricted to the deepest part of lamina II and were relatively numerous in lamina III, thus differing in their distribution from the PPD cells. None of the 324 PPD cells examined in these sections was parvalbumin-immunoreactive (Figure 6d-f). NPY-immunoreactive cells were found throughout laminae I-III, however, few of the PPD cells examined (21/308, 6.8%) were double-labelled (Figure 6g-i). Three of the double-labelled cells were located in lamina I and 18 in lamina II, and all of them were weakly stained with the NPY antibody.

**Figure 7:**
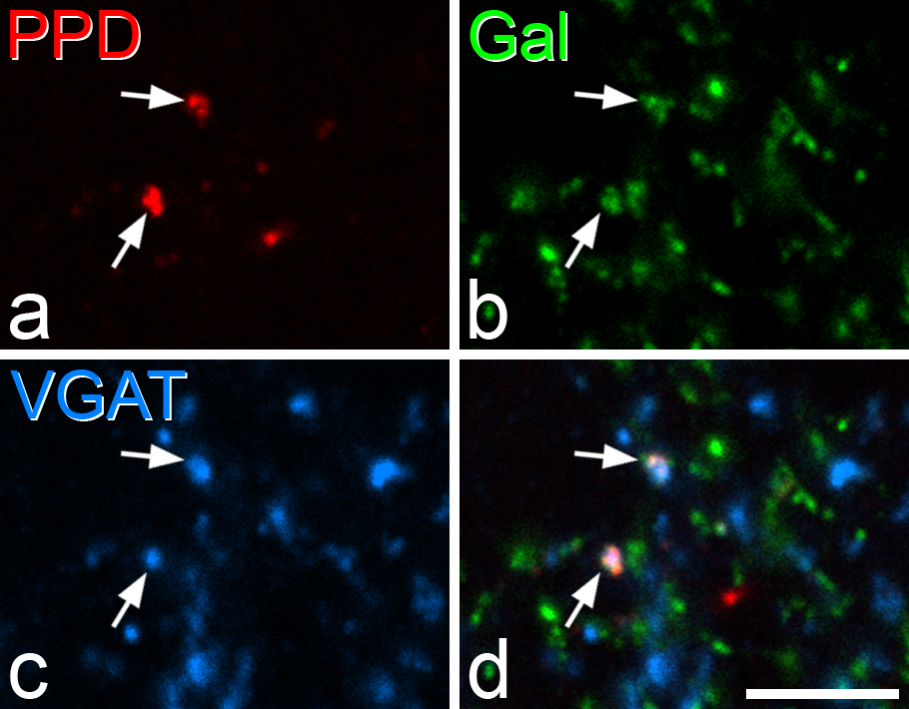
**Co-localisation of PPD and galanin in GABAergic boutons**. **a-c**: Confocal images scanned to reveal PPD (red), galanin (Gal, green) and VGAT (blue). **d**: a merged image. This field from lamina IIo contains two boutons (arrows) that are immunoreactive with all 3 antibodies. Note that several boutons that are immunoreactive only for galanin are also present, and these are likely to belong to peptidergic primary afferent axons, which also contain galanin. The images are from a single optical section. Scale bar = 5 μm.

In contrast, there was extensive co-localisation of PPD and galanin in neurons in the superficial dorsal horn (Figure 6j-l; Table 5). In lamina I, 47% of the PPD neurons were galanin-immunoreactive, and double-labelled cells constituted 96% of the galanin-containing population. In lamina II, 70% of the PPD cells were galanin-positive, and double-labelled cells made up 92% of those that contained galanin. Although scattered PPD^+ ^and galanin^+ ^cells were observed in deeper parts of the dorsal horn (laminae III-IV) in these sections, none of these were double-labelled. There was also extensive co-localisation of PPD and galanin immunoreactivities in VGAT boutons in the superficial dorsal horn (Figure 7). Among the PPD^+^/VGAT^+ ^boutons, 93.3% (range 92-94%, n = 3 rats) were galanin-immunoreactive, while 91.3% (range 91-92%) of the galanin^+^/VGAT^+ ^boutons contained PPD.

## Discussion

The main findings of this study are: (1) that cells expressing PPD are relatively common in the superficial dorsal horn, constituting 17% of neurons in lamina I and 4% of those in lamina II, (2) that the majority of these (69%) are GABAergic, and (3) that among the neurochemical populations of inhibitory interneurons that have been identified in the rat dorsal horn [47,64], the PPD cells overlap extensively with those that contain galanin, but not with those that express nNOS, NPY or parvalbumin.

### Dynorphin-expressing neurons

The concentration of dynorphin in the cell bodies of neurons that synthesise the peptide is normally too low to be detected by immunocytochemistry, either because dynorphin is rapidly transported away from soma to axon terminals, or because it is only produced from PPD within the axon. Most of the early studies that investigated the distribution of dynorphin-containing cells in the spinal cord therefore used colchicine to block axoplasmic transport and increase perikaryal labelling [26-32]. However, it is now known that colchicine can alter the mRNA levels for neuropeptides [75-77], and may even cause *de novo *synthesis of these peptides by neurons that do not normally express them [75,77]. In order to avoid the need for colchicine, we used an antibody against the precursor protein, PPD, which can be detected in neuronal cell bodies in both brain and spinal cord [35,46]. It has also been shown that PPD and dynorphin are co-localised in axon terminals in the dorsal horn [35], indicating that PPD antibody can be used to reveal the axons of dynorphin-containing cells. The distribution of PPD-immunoreactive neuronal cell bodies that we saw is the same as that reported by Marvizon et al. [35], who used a different PPD antibody and also closely resembles that of PPD mRNA^+ ^cells detected with *in situ *hybridisation in rat [31] and mouse [36]. In addition, the laminar location of PPD-immunoreactive axons was very similar to that of dynorphin-containing axons reported in various species in previous immunocytochemical studies [25,28,33-35]. Many of these axons are likely to originate from local PPD-expressing cells, however, some are of primary afferent origin [35]. In addition, the presence of PPD in the dorsolateral funiculus raises the possibility that there are dynorphin-containing axons originating in the brainstem that project to the spinal cord.

Although the majority of the PPD^+ ^neurons in the superficial dorsal horn were GABA-immunoreactive, and were therefore inhibitory interneurons, half of those in lamina I and 20% of those in lamina II were not stained with the GABA antibody, and these were presumably glutamatergic (excitatory) cells. This interpretation is consistent with the finding that PPD is co-localised with VGLUT2 in some axons within the superficial dorsal horn [35]. The glutamatergic PPD cells in lamina I are likely to include projection neurons. Although the early studies that demonstrated dynorphin in lamina I spinoparabrachial neurons [30,32] used colchicine, which may have resulted in *de novo *expression of the peptide, Li et al. [39] reported that ~7% of lamina I trigeminothalamic neurons were PPD-immunoreactive. Marvizon et al. [35] did not detect PPD staining in the cell bodies of neurons with the neurokinin 1 receptor, which is found on ~80% of lamina I projection neurons [78], and it is therefore possible that dynorphin expression is restricted to those projection cells that do not possess this receptor.

Little is known about the types of excitatory interneuron that contain PPD. Several neurochemical markers for subsets of excitatory interneurons have been identified, including neuropeptides (e.g. somatostatin and neurotensin), neuropeptide receptors (e.g. the μ-opioid receptor MOR-1) and other proteins (e.g. PKCγ). Marvizon et al. [35] reported that PPD was not present in cells that were labelled with antibodies to either MOR-1 or PKCγ. The lack of overlap with PKCγ is not surprising, since PKCγ-expressing neurons are concentrated in lamina IIi and lamina III, where PPD neurons were very scarce. However, MOR-1-immunoreactive cells, which make up around 10% of all lamina II neurons [79], are found throughout the depth of the lamina [80], and the lack of coexistence of MOR-1 with PPD therefore suggests that dynorphin is expressed by specific types of excitatory interneuron.

Dynorphin-containing neurons in the spinal cord show considerable plasticity. The peptide can be strongly up-regulated in both superficial and deep parts of the dorsal horn in various pain states [31,37,38]. Further studies will be needed to determine whether these changes affect both excitatory and inhibitory neurons in the superficial laminae.

### Subpopulations of inhibitory interneurons in the superficial dorsal horn

We have previously identified four distinct populations of inhibitory interneurons in laminae I-III, based on the expression of different neurochemical markers: NPY, galanin, nNOS and parvalbumin [47,64]. Although all NPY and galanin cells in this region are GABAergic [69,81], some of the cells that contain parvalbumin [64,82] or nNOS [61] are non-GABAergic. These populations of inhibitory interneurons have characteristic laminar distribution patterns: those that contain galanin are highly concentrated in lamina I and IIo, the parvalbumin cells are largely restricted to the inner part of lamina II and lamina III, while cells that contain NPY or nNOS are found throughout laminae I-III. Although there is a small group of neurons in lamina III that contain both galanin and nNOS, apart from this there is virtually no co-localisation of any of these compounds within neurons in this region, indicating that these four markers can be used to define distinct, non-overlapping populations of inhibitory interneurons [47]. We have also estimated that cells belonging to these four populations account for around two thirds of the inhibitory interneurons in lamina I, and half of those in lamina II [61].

The present results demonstrate that half of the PPD cells in lamina I and the great majority (80%) of those in lamina II are GABAergic, and that these cells are largely equivalent to one of the neurochemical classes that we have previously defined: those that contain galanin. In both lamina I and lamina II, the proportion of PPD neurons that contained GABA (47% and 79%, respectively) and the proportion of those that contained galanin (47% and 70%) were very similar, and the great majority of the galanin cells in both laminae were PPD^+^. Taken together with the fact that all galanin cells are thought to be GABAergic [81], these findings indicate that dynorphin-expressing cells in the superficial laminae can be divided into 2 main groups: a population of PPD^+^/galanin^+ ^inhibitory interneurons, which are present throughout laminae I and IIo, and a smaller population of PPD^+^/galanin^- ^excitatory neurons, most of which are located in lamina I.

PPD is present in 17% of the neurons in lamina I and 4.3% of those in lamina II, and since 47% and 79%, respectively, of these are GABAergic, we estimate that PPD^+^/GABA^+ ^cells constitute 8% of all neurons in lamina I and 3.4% of those in lamina II. We have previously reported that in the L4/5 segments of Sprague-Dawley rats, GABAergic cells make up 24.8% of the neuronal population in lamina I and 31.3% of that in lamina II [3]. If we assume that the proportions are similar in Wistar rats, then PPD would be present in ~32% of the inhibitory interneurons in lamina I and 11% of those in lamina II. We have previously estimated that galanin-containing cells (which overlap extensively with the PPD population) account for 26% and 10% of GABAergic neurons in laminae I and II, respectively [47].

As expected from the extensive co-localisation of PPD and galanin in cell bodies, they were also co-localised in most GABAergic boutons that contained either peptide. Interestingly, both PPD and galanin were found in a relatively small proportion of GABAergic boutons in laminae I and II: ~4% in both laminae for PPD (present study) and 6 and 3%, respectively, for galanin [47]. Since cells that contain these peptides make up a far higher proportion of the GABAergic cells in these laminae, this suggests that these neurons may generate relatively small axonal arbors compared to other types of inhibitory interneuron in this region, and that they are therefore under-represented among GABAergic axons within the superficial dorsal horn.

Ross et al. [5] have recently shown that mice lacking the transcription factor Bhlhb5 develop skin lesions that are thought to result from a heightened sensation of itch, and that this behavioural phenotype depends on the loss of a set of dorsal horn inhibitory interneurons that normally express Bhlhb5. They also reported that in wild-type mice the transcription factor could be detected in restricted populations of inhibitory and excitatory neurons for up to two weeks after birth. We have found that in neonatal mice PPD-immunoreactive cells have a similar distribution to that seen in adult rats, with numerous cells being present in the superficial dorsal horn, and that virtually all of the PPD^+ ^cells express Bhlhb5 (E. Polgar, A.J. Todd and S.E. Ross, unpublished data). This raises the possibility that the dynorphin-expressing inhibitory interneurons may play a role in the prevention of itch.

## Conclusions

The neuronal organisation of the superficial dorsal horn is extremely complex, and still poorly understood, and this is largely due to the difficulty in defining discrete populations of inhibitory and excitatory interneurons. The present results provide further evidence that a neurochemical approach can be used to identify distinct classes of inhibitory interneurons, by showing that dynorphin is largely restricted to those cells that also express galanin. The ability to identify the axons of these cells by dual immunolabelling for VGAT with either PPD or galanin will allow the postsynaptic targets of these cells to be determined, and this should help to elucidate their role in the synaptic circuitry of the dorsal horn.

## List of abbreviations

NA: numerical aperture; nNOS: neuronal nitric oxide synthase; NPY: neuropeptide Y; PKCγ: protein kinase Cγ; PPD: preprodynorphin; TSA: tyramide signal amplification; VGAT: vesicular GABA transporter

## Competing interests

The authors declare that they have no competing interests.

## Authors' contributions

TCPS and EP participated in the design of the study, the experiments and the analysis; FG participated in some of the experiments; MW, TF and TK generated antibodies used in the study; AJT conceived of the study, participated in the design, experiments and analysis, and drafted the manuscript. All authors contributed to the writing of the manuscript and approved the final version.
